# Automated Surveillance of Fruit Flies

**DOI:** 10.3390/s17010110

**Published:** 2017-01-08

**Authors:** Ilyas Potamitis, Iraklis Rigakis, Nicolaos-Alexandros Tatlas

**Affiliations:** 1 Department of Music Technology & Acoustics, Technological Educational Institute of Crete, Rethymno Crete 74100, Greece; 2 Department of Electronics, Technological Educational Institute of Crete, Chania Crete 73133, Greece; rigakis@chania.teicrete.gr; 3 Department of Electronics Engineering, Piraeus University of Applied Sciences, Athens 12244, Greece; ntatlas@puas.gr

**Keywords:** precision agriculture, insect surveillance, automatic monitoring

## Abstract

Insects of the Diptera order of the Tephritidae family cause costly, annual crop losses worldwide. Monitoring traps are important components of integrated pest management programs used against fruit flies. Here we report the modification of typical, low-cost plastic traps for fruit flies by adding the necessary optoelectronic sensors to monitor the entrance of the trap in order to detect, time-stamp, GPS tag, and identify the species of incoming insects from the optoacoustic spectrum analysis of their wingbeat. We propose that the incorporation of automated streaming of insect counts, environmental parameters and GPS coordinates into informative visualization of collective behavior will finally enable better decision making across spatial and temporal scales, as well as administrative levels. The device presented is at product level of maturity as it has solved many pending issues presented in a previously reported study.

## 1. Introduction

This work presents our most recent results on an electronic trap for automated monitoring of insect populations of fruit flies (Diptera of the Tephritidae family). Fruit flies each year cause worldwide crop losses calculated in billions of euros [1]. Female *Bactrocera oleae* (Gmelin), *Ceratitis capitata* (Widemann), and *Bactrocera Dorsalis* (Hendel), among many other species, demonstrate high reproductive rates. These pests lay eggs beneath the fruit surface, these eggs hatch and the larvae feed inside the fruit. As a result, the fruit drops or its quality degrades. Pests can be controlled with ground pesticide sprays or biological means, the efficiency of which depends on knowing the time, location and extent of infestations as early as possible. The decision on when to begin treatment is usually based on manual crop scouting. Insect traps attract the pest using chemical signals that originate from pheromones or food baits placed inside the trap. A network of monitoring traps can be composed of tens to hundreds of traps and, in large orchards, can be dispersed with many miles between nodes. Automatic monitoring of pests in agricultural crops in the context of our work intends to provide the following features:

(a) Increased productivity due to timely delivery of comprehensive information to a central agency. The central agency receives information on the location and density of the targeted pest as well as microclimate parameters. This information can be used to alert for the presence of the pest and serve as supportive evidence to initiate treatment procedures. The onset of an infestation is a crucial parameter that is often missed in manual inspections as it may fall between scheduled manual visits. Traps are visited every 5 days. In this time period the amount of *B. oleae* insects inside the trap would not exceed 20, because the Economic Injury Level for *B. oleae* is 5–20 insects per trap in a 5 days period. Economic Injury Level in pest management means that if you miss this point then economic damage begins and in the case of this pest it can be large.
(b) Time-stamping of the event of insect entrance in the trap allows the gathering of precise information on the life cycle of the pests and their relation with different pheromone and/or food baits. Moreover, it allows continuous and real-time evaluation of the results of applied treatments. The central monitoring agency securely reflects the current situation of the infestation and not a situation that has evolved to an unknown state due to delays in the delivery and interpretation of the relevant information.
(c) Reduction on the application of pesticides and increase of their applied efficiency. Cultivators often start treatment too early or overspray for fear of missing the infestation onset. Knowledge of where and when to apply a treatment can mitigate the problem of over-application of pesticides in one region and under-application in another.
(d) Increase the profit margin by decreasing the current labor-intensive and expensive manual monitoring activities. Current manual inspection involves field scouters visiting a remote network of traps on a regular basis. This procedure entails a time lag between the phenomenon and its report, and increases the cost due to transportation expenses and wages.
(e) For countries where fruit production represents a significant percentage of the total gross income, monitoring and control is handled by the state, while agricultural unions and large orchard owners can take further actions. Once the trap is located, the human observer must discern and count the targeted pests. This is not always feasible as these may have disintegrated or be obscured in a maze of insects. This practice is a complicated procedure that involves open tenders, contracts, qualified personnel working at different report layers. Because of the number and location of the traps, the frequent trips and the expertise required, the requirements of the monitoring protocol are compromised in practice, as one may judge from the large reported losses due to Diptera infestations.

The penetration of high-end technology in the automated identification of insects and possibly control of pests has occurred and is gaining solid ground [2,3,4], but is far from being considered established in view of cost-effective and practical applications adopted by cultivators. In [5,6,7,8,9] researchers tried to sense the presence of a targeted pest and report counts for various insects of interest. The vast majority of reported cases treat the insect as a falling body that causes a photo-interruption which leads to a binary decision and counting.

In the early, inspiring work reported in [10], the authors presented a basic optical tachometer for measuring the wing-beat frequency of free-flying insects. This work, as well as several works inspired by it (e.g., a 2D-receiver version of [10] is shown in [11]), demonstrated the basic idea of a sensor recording the modulated light produced by the flapping wings of insects. This early work however, did not deal with practical issues that need to be addressed in order to produce a device capable of working efficiently either in the controlled environment of a laboratory or in the open field. In a normal laboratory there are many electronic appliances that cannot be simply switched off. Insectaries normally breed insects under automatic illumination conditions that mimic the daily light cycle and use electrical devices that maintain certain levels of humidity and temperature. Fluorescent lamps and tubes, among other types of lamps, can cause two kinds of problems: (a) they can function as light emitters that can override the light-emitting diodes (LED) lights of the sensor and (b) they produce electromagnetic interference that starts from the frequency of the mains supply and their strong harmonics can reach several thousands of Hz, thus corrupting the sensitive measurement of a wing-beat. In [12,13] we presented the details of a practical way to deal with this specific problem by modulating the insect wingbeat signal to very high-frequencies, cleaning the lower frequencies contaminated with all sorts of interferences and demodulating back the insect’s frequencies. The work in [12,13] deals with the problem of electromagnetic and light interferences and assumes unlimited access to power. In [14] we reported on an attempt to develop field applications of the technology by presenting an electronic insect trap that counts and identifies insects. As reported in [14] (Conclusions section, p. 23) that electronic trap had several efficiency and power consumption limitations. When operating in orchards, the main problem is not electromagnetic interferences from electrical devices as in [12,13] but the limited power resources. The work presented here overcomes these constraints, including the power efficiency barrier, and offers a description of a product-level implementation. To achieve this we introduce several distinct novelties in the construction of sensors and electronics to shift the research on this device from good science to good practice. In detail:

(a) The receiving aperture of the sensor is made large enough to allow tracking of fast flying insects such as fruit flies that would otherwise spend little time inside the field of view (FOV) while crossing the surface of a single photodiode. Lack of sufficient duration data is translated into poor frequency resolution for fast flying insects such as fruit flies.
(b) Our boards were redesigned based on low-power electronics and optimized software to maintain power consumption at a sufficiently low level in order to be able to operate the device in the field for at least two months without the need for a solar panel.
(c) Our new algorithmic design based on interrupt-driven circular buffers never misses the onset of the wingbeat, even when it occurs before the initialization of the recording process.

In the near future, provided that automated monitoring will reach large scale deployment of the monitoring nodes, the correlation of insect counts with environmental parameters such as humidity, temperature and global positioning system (GPS) coordinates will allow the prediction of food productivity and prices using statistical predictive modeling. Moreover, the transmitted results can be correlated and co-interpreted along with the general state and changes of the climate. Prediction of future infestation outbreaks based on historical data will also be feasible. Finally, the traps can serve as beacons for summoning and guiding unmanned flying vehicles (drones) to spray only in designated areas. Therefore, for large agricultural areas the repetitive circle of treatment with spraying drones and after-treatment assessment with e-traps can be automatized to a great extent.

## 2. Materials and Methods

### 2.1. Internal Configuration

**Figure 1:**
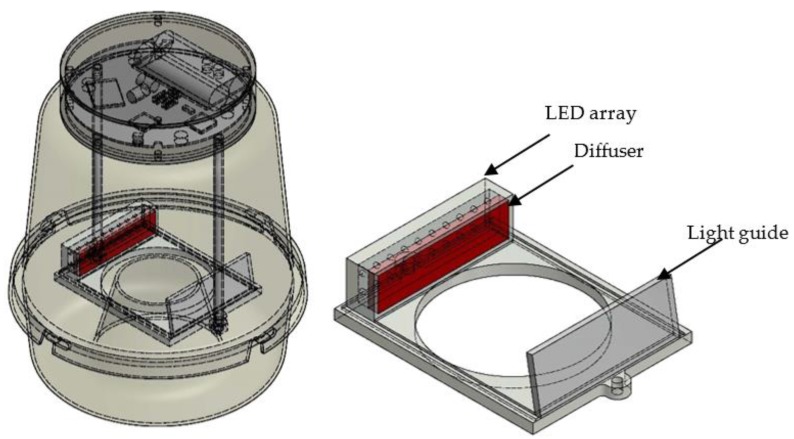
Schematic diagram of the automated monitoring trap (**a**) and enlarged details of the optical sensor (**b**).

**Figure 2:**
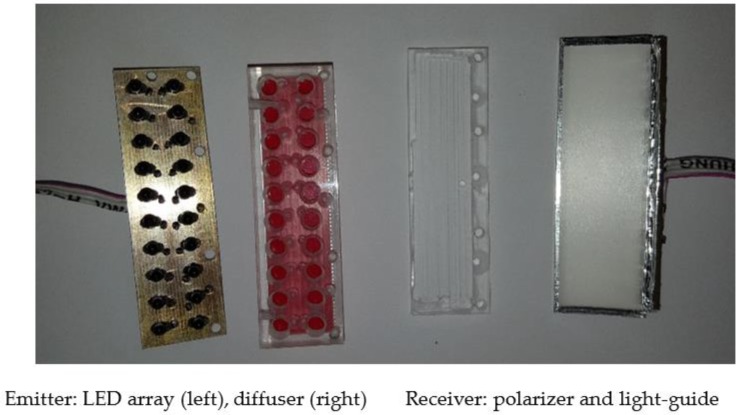
Sensor. The first two items on the left compose the emitter and the other two on the right, the receiver.

The device is designed so as to introduce the minimal disturbance into the internal space of a McPhail type trap. We build on traps that have been proven in time to be compatible with the life cycle of insects as we did not want a new design to jeopardize the effectiveness of the trap in attracting insects. Therefore, all electronics are gathered in a slim 2.75 cm thick add-on component attached from the outside on the top of the trap (see Figure 1a), and all plastic parts inside the trap are made transparent. The size of the electronics, compared to [14], is halved without any compromise in the processing capabilities or its cost. All cables are directed inside and through the supporting tubes that also serve as holders of the sensor that monitors the entrance to the trap. There is an intentional gap between the internal border of the inverted funnel and the holder of the sensor as fruit flies may enter in two ways: either directly flying-in or landing outside, walking to the inner border and then flying in as there is no other way to enter the trap. The insects follow the high concentration of odors at the top of the trap due to bait evaporation that is placed at the bottom and stick to the walls of the trap as these are better illuminated than the interior. The trapped insects finally fall in the bait out of exhaustion or because the bait contains detergent that evaporates along with it. The reverse motion of flying out is atypical for the flight patterns of the fruit-fly (see video supplement) and the shields around the sensor protect the sensor from double counting. Regarding the optical sensor seen in Figure 1b, we use a light guide as a receiver and an array of infrared LEDs with an attached diffuser as an emitter (see Figure 2). A 1D linear array as well a 2D array of photodiodes proved insufficient for flying insects such as fruit flies, as their fast movement and relatively low wingbeat frequency did not leave enough traces for their efficient identification. The photodiodes we embedded have 3.5 mm width and the gaps between them effected a variation in the intensity of received light as the fruit fly crossed the diode in less than 30 ms. The light guide with a wide receptive area of 7.2 × 2.3 cm^2^ has solved all problems of this kind and delivers an average 100 ms of wingbeat recordings. The light guide is made of parts of laptop screen sheets and is used in reverse functionality. In laptop screens the light is directed from the motherboard up the screen and out whereas, in our case, the infrared light from the emitter placed at the opposite to the light guide is projected down to the base of the screen where it is collected by a 1D array of photodiodes attached to one edge of the light guide. Note that the light guide is not functioning as a screen and the array of photodiodes is only collecting fluctuations of light intensity.

Diffusers and polarizers (see Figure 2) are commonly used in thin-film transistors and laptop screens. In the context of our work, the diffuser is used so that the light guide sees a smooth light distribution of almost equal intensity, stemming from a rectangular array of the LED array. The polarizer improves the SNR of the signal in the receiver by almost 3 dB. The waveguide and polarizer are similar to these used in laptop screens (type LTN154U2-L03 Samsung street, Asan, South Korea). We chose to not apply Fresnel lenses, or other lenses, in order to keep down the cost. Moreover, these lenses require a certain focal distance and that would make the sensor design be bulkier. We cannot jeopardize the practicality of the trap by occupying its internal space and the light guide proved very slim and cost-effective.

The number of LEDs was defined by a trial and error procedure. Fewer LEDs lead to areas with higher light intensity than others. This results to variations in received signal power as a fast insect passes from the lighter to the less illuminated spot and back. This is also more visible when one employs 2D arrays of photodiodes. To secure that the receiver senses the same light intensity at the borders, the LEDs around the borders are driven with higher current than the central ones as otherwise the receiver would receive a greater intensity of infrared light in its center. We use photodiodes (part No. TEMD5110Χ01, Vishay Intertechology, Inc., Malvern, PA, USA) that have a peak sensitivity at 940 nm and 90% of peak sensitivity at 850 nm. Although there are LEDs matching these photodiodes (e.g., SFH4346, OSRAM Opto Semiconductors GmbH, Regensburg, Germany) we chose another LED, the SFH4356, with a transmitting wavelength 850 nm, but with a smaller rising time as a small rising time means that the LEDs need to remain activated for much shorter time and thus we could cut power consumption. More details on the power consumption and how this can be extended for the desired timespan, will be provided. In this work, the plastic trap is covered with a sun-blocking film (Crystalline CR70, 3M, Saint Paul, MN, USA) that allows visible light to enter the trap but, according to the specifications, it rejects 97% of infrared radiation at 900–1000 nm.

### 2.2. Electronics

The trap is designed to be operated in the field without a constant power source. The main issue of concern during development was how to implement a device with as low power consumption as possible. Because of the limited power resources one has to set priorities for performed tasks and algorithmic complexity. First, we give details on the power consumption plan and how this can last for the desired duration. We used a MSP432P401R microprocessor (MCU, Texas Instruments, Dallas, TX, USA) to carry out all tasks, from sending pulses through the emitter and monitoring the light intensity at the receiver to process the recordings and delivering data through the GPS-GRPS card (see Appendix A for details). Power consumption totals 9.57 mW of which 4.3 mW are consumed by the analogue part of the device (LEDs 4.29 mW, photodiodes amplifier 10 μW) and 5.28 mW in the digital part (MCU 4.95 mW, SD 0.33 mW). The device is powered by two 3.7 V batteries (2 × 3000 mAh) and when following a report schedule of once per day and a typical case of 10 triggers per day the device has sufficient power for 60 days.

**Figure 3:**
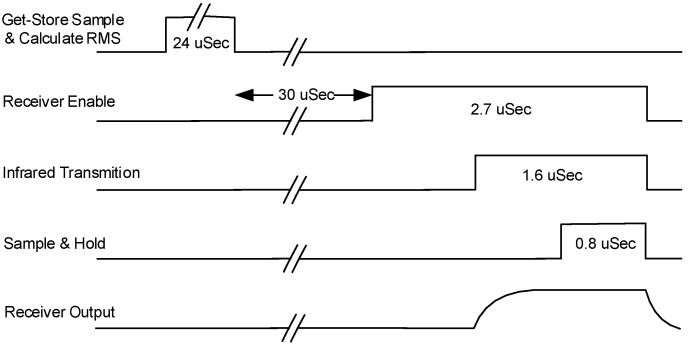
Synchronization diagram between emitter and receiver. The process is repeated every 250 μs. The MCU is only active during the Store Sample & Calculate root-mean squared (RMS) step.

The emitter sends pulses to a synchronized receiver (i.e., intermittent light as opposed to continuous light). These pulses have large amplitude but very short duration. Their brevity means that the emitting LEDs need to be powered only for a small fraction of time and therefore we make our electronic design power efficient. The clock of the microprocessor-unit (MPU) synchronizes the emitter and receiver to be switched on and off repeatedly and simultaneously and, therefore, the MPU does not consume computational resources. The pulse train arriving to the receiver is driven to an envelope detector that keeps only the difference in amplitude from pulse to pulse. Since we track the amplitude variation from pulse to pulse and not the pulse amplitude *per se*, the sensor is immune to slowly varying changes in illumination conditions (i.e., the pass of a cloud, daily cycle of light etc.). Hereinafter, we give more detail to this elaborate process:

The clock of the MPU instructs the emitter to send high energy pulses of 200 mA with duration of 1.6 μs every 250 μs (i.e., at a frequency of 4 kHz). Pulses are transmitted and received in accordance to the clock. The synchronization pulses are produced by the embedded timer of the MPU in capture/compare blocks. The duration of the pulses has been defined from the response time of the photodiodes (500 ns). Hereinafter, we describe a period of 250 μs (see Figure 3):

1. ADC process and data storage
2. After 24 μs, enable receiver
3. At 1100 ns, emit a 1.6 μs pulse
4. At 800 ns, Sample—Hold for 800 ns
5. De-activate emitter, de-activate receiver.

The output signal from the receiver is driven to a sample & hold circuit. Subsequently, it is driven through alternating current (AC)-coupling to an amplifier so that one amplifies the useful signal, and then to the embedded analog-to-digital converter (ADC) of the processor. Non-continuous light is the key to low-power operation. It should be noted that no solar cell is required and the device can last longer if it is switched off at night as *B. oleae* is only active during daylight. The Global System for Mobile Communications (GSM) is always in sleep-mode. It is woken up by the MPU on a prescheduled basis, transmits and is switched off again. During GSM activation-transmission all other signal processing procedures are halted to avoid electromagnetic interference. Transmission takes place at night when *B. oleae* is inactive; therefore, no loss of wingbeat events of the target pest are possible.

**Figure 4:**
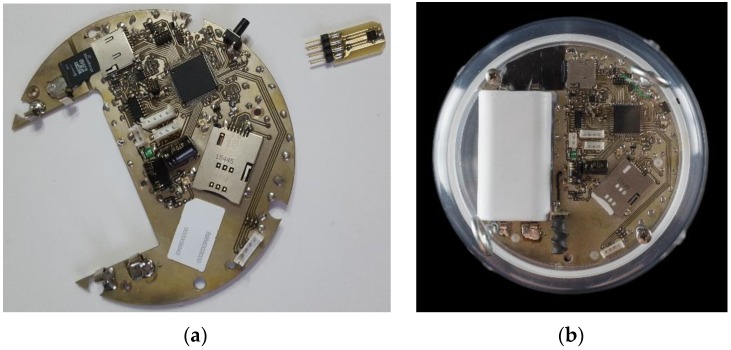
The electronic board of the automated fruit fly trap (**a**) and final placement of the board in the trap (**b**).

The signal to noise ratio (SNR) than can be achieved by a 14 bit ADC is in theory approximately 86 dB. The 14 bit depth of the embedded ADC of the processor is enough to code the analogue signal of the receiver as the noise level of our system is −75 dB. Therefore quantization noise is lower than the produced noise. The electronic board and associated environmental sensors are shown in Figure 4a and their final placement in Figure 4b.

### 2.3. Code

The embedded microprocessor runs a constantly-looping program which processes the data captured by the optical sensor. The board is programmed in optimized C/C++. The MCU executes two basic procedures: The first one is interrupt-driven and stores the ADC data in a circular buffer of 16 K samples. Therefore, the line-level output from the optoelectronic sensor is copied to a circular buffer. The same procedure monitors the signal’s root-mean-square (RMS) using a window of 128 samples (16 ms in 4 kHz sampling rate). If the RMS of the window exceeds a predefined threshold, an event has been detected, i.e., an insect has crossed the sensor’s FOV. This triggers the recording of the signal capturing 1024 samples of 256 ms duration from the second cyclic buffer coded with 14-bit resolution, at 4 kHz sampling rate. There is no further processing or storage prior to the triggering event excluding the circular buffer. After triggering, 200 samples (i.e., 50 ms) are drawn before and up to the triggering point and 824 samples (i.e., 206 ms) after that point in order to ensure that the onset of a wingbeat event is not lost. The low sampling frequency is enough to sample the wingbeat frequency of fruit flies expected around 200 Hz and several of its partials as well. Wingbeat events are short in time for fast flying insects such as flies and one cannot afford discarding any useful part of the signal such as the onset. The sampling frequency, window length and triggering threshold are pre-stored in the secure digital (SD)-card and are read once during powering-on of the device. After triggering, the subsequent performed tasks are: (a) Fast Fourier Transform (FFT) of the data chunk captured; (b) decision on the identity of the insect based on analysing its wingbeat and (c) storing the wingbeat snippet in the SD card and queuing information to be transmitted on a pre-scheduled basis to a server. If during the FFT process we have a new event this will be stored in the main 16K buffer and will be served when the MCU completes the previous event. When there is no trigger, all MCU procedures but the window monitoring the RMS level rests in sleep mode. The exact duration of the processing stages in analysed in Table 1:

## 3. Results

**Figure 5:**
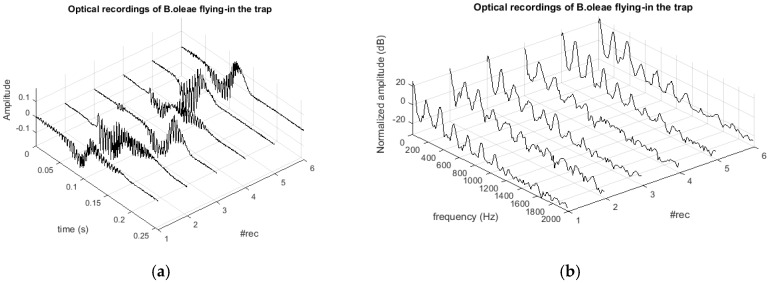
(**a**) Optical recording of different cases of *B. oleae* flying in the trap. High-frequency modulation due to wingbeat and low-frequency main-body movement; (**b**) spectra of the corresponding recordings. The fundamental frequency is at around 200 Hz and at least five harmonics are resolved.

The triggering stage results in a data chunk of 1024 samples containing only the wingbeat event. In Figure 5 we show examples of the wingbeat events. There is a slow varying in time amplitude part (see Figure 5a), almost non-oscillatory, which is due to the main body movement that appears at low frequencies near DC in Figure 5b. We would get the same slowly varying pattern with a simple drop of an object through the FOV. On the slow varying part one can clearly see the imposed oscillatory contribution due to the modulation that the wingbeat inflicts on the light intensity. The power spectral density (PSD) one-sided estimate of each snippet is found by splitting the waveform in 4-chunks of 256 samples each without overlapping. The modified periodogram is computed using a Hamming window of 256 samples followed by a 256 points Discrete Fourier Transform (DFT). The DFT’s are averaged and log-transformed to obtain the PSD estimate of the recording. In Figure 5 we line-up several wingbeat recordings from different *B. oleae* individuals taken from the trap in operational mode and stored in the embedded SD. The SNR is around 50 dB calculated as:

(1) $$SNR\;=\;20\;\times \;log\left(\frac{Signal\_RMS}{Noise\_RMS}\right)$$

We use the frequency content of the wingbeat as biometric evidence for species classification. Note that, besides the fundamental frequency, information bearing parts of the signal are the exact location of the partials (some higher partials can be detuned from the exact placement of harmonics), the distribution of energy on the harmonics, and the amplitude of the near-DC frequency content related to body-size. While the aforementioned spectral areas are the most informative, species identity information is distributed in all over the spectrum. This is a common situation as in the speaker and language identification tasks in speech processing, therefore the use of the whole spectrum is suggested as opposed to estimating the f0 and the harmonics solely.

### 3.1. Verification Results in the Lab

**Figure 6:**
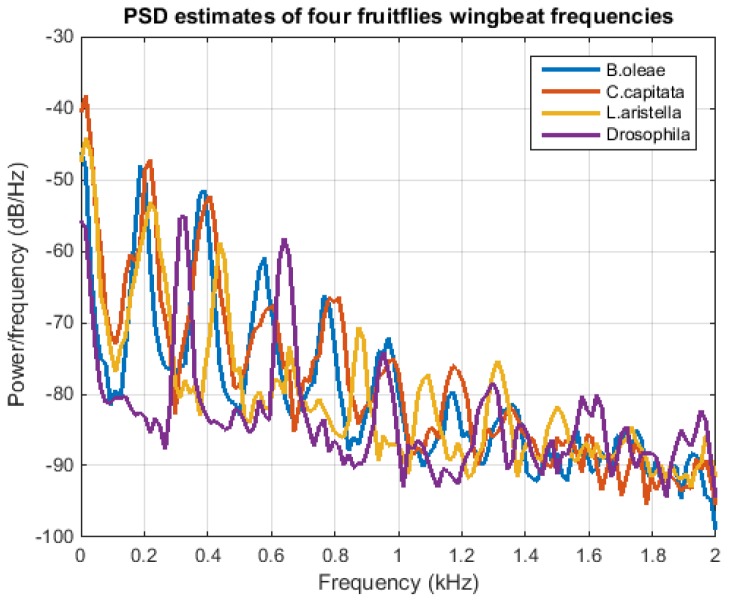
Power Spectral Densities of the wingbeat of four fruit flies. The fundamental and the harmonics overlap significantly.

Verification involves answering a problem with a binary answer: once the device is triggered, due to detection of an entering insect, the question is whether then becomes was it the *target species* (i.e., *B. oleae* in our case) that flew in or not. We are not interested in the context of this work to recognize any species other than the target pest. Two approaches were examined: (a) a simple, rule-based approach that reports results based on calculations carried out inside the trap, and (b) results based on analyzing off-line the stored recordings. The first approach is based on two simple rules that simultaneously must hold in order to count a signal as a target case: *Rule 1*: Examine if the amplitude of the time domain recording is within the limits derived from a large number of *B. oleae* recordings (see Figure 5). Insects that have larger wings than *B. oleae* will return a larger fluctuation on signal amplitude and, on the contrary, insects that are much smaller (e.g., midges) modulate light at much smaller amplitudes. *Rule 2*: Examine the frequencies between 170 and 230 Hz where the fundamental frequency of *B. oleae’s* wingbeat lies and if the energy of this bandwidth exceeds a threshold then call it a verified detection. Thresholds are derived from positive examples in the lab. The rule is simple and efficient provided there are no competing fruit-flies in the olive orchard. This rule cannot, by default, tell the difference between *B. oleae, C. capitata* and *Lonchaea aristella* (Diptera: Lonchaeidae) whose spectra totally overlap (see Figure 6).

Nevertheless the approach rejects 80% of *Drosophila* cases and 100% of all mosquito cases we tried. *Drosophila* is a smaller insect compared to *B. oleae* and beats its wings at higher frequencies between 260–310 Hz and mosquitoes have an even higher fundamental frequency when all are observed in the same reference temperature. We never observed triggering of the device in the absence of an insect flying in. Similarly, the device is immune to insects walking without flapping their wings. The rule-based version of examining the spectral area around 200 Hz is suggested to be applied with sex-specific pheromone attractants to avoid attraction of non-target insects. We then focused on employing more complex and computationally involved algorithms that currently must be executed out on a server to examine the full information quality of the wingbeat. In practice this would entail the transmission of the snippet from the SD of the trap to the server. 

**Figure 7:**
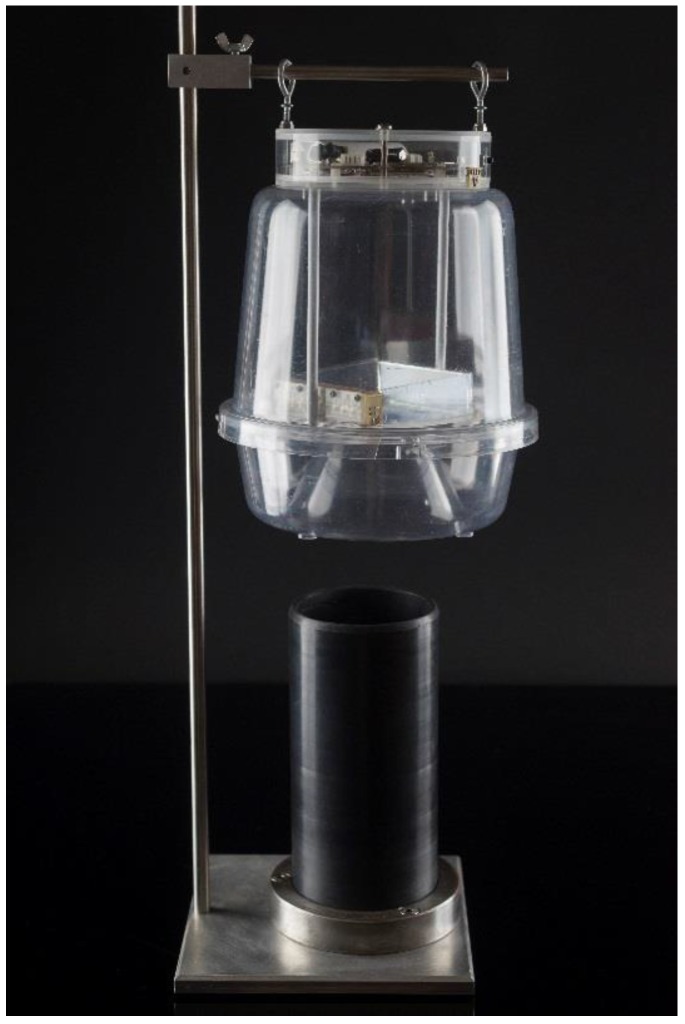
Testing setup. The trap is fixed on the entrance of a dark tube. Fruit flies are placed inside the tube. Insects follow the light at the end of the tunnel and either fly in the trap directly or most commonly, walk until the internal border of the trap and then fly in.

All recordings in Table 2 are taken by insects that either flew in from the container to the trap or landed on the outside of the inverted funnel entrance, walked inside up to the border that they tend to explore by walking and then flew in (see Figure 7 for the testbed). No attractant was used other than physical light and random variation in temperature and humidity was kept as low as possible. One should note that this is a challenging dataset reflecting a worst case scenario in reality as it investigates the possibility of discerning a specific fruit fly among other fruit flies only from its wingbeat. 

The recorded dataset depicted in Table 2 is based on the experimental setting shown in Figure 7. All adult insects of all species in this work started as larvae inside fruits (i.e., olives for *B. oleae*, peaches for *C. capitata* and figs for *L. aristella*) and they feed upon the pulp until they exit, usually as third instar larvae. Then they are collected and grown in an insectary cage. As larvae turn into adult insects, we supply them with yeast hydrolysate-sugar diet and water to sustain them to life. We keep only first generation insects, as breeding generations of insects in captivity results into degeneration that might affect the flying mechanism and our target are insects of the real-field. Each insectary cage contains strictly one species and about 200–300 adults of both sexes. 

Adult insects are taken in turn and placed in the black container until they fly into the trap. All *B. oleae* snippets are tagged with label “1” and all others with label “0”. We first assessed the verification performance of popular classifiers based on 10-fold cross-validation. The whole dataset is randomly shuffled, 80% of the dataset is used for training and the rest 20% for testing. The whole procedure is repeated 10 times and the mean accuracy and standard deviation over 10-folds is reported in Table 3. To further elaborate on the recognition accuracy we use precision, recall and F1 score metrics on a random 20% holdout part of the dataset and also derive the confusion matrix of the test set. One should note that in binary classifications there are two sources of errors: the system can fail to classify correctly a target (i.e., a miss) and the system can erroneously classify a fruit fly that is not *B. oleae* as such (i.e., a false alarm). Precision (P) is defined as the number of true positives (*Tp*) over the number of true positives plus the number of false positives (*Fp*):

(2) $$P=\frac{T_{p}}{T_{p}+F_{p}}$$

Recall (*R*) is defined as the number of true positives (*Tp*) over the number of true positives plus the number of false negatives (*Fp*):

(3) $$R=\frac{T_{p}}{T_{p}+F_{n}}$$

These quantities are also related to the (*F*_1_) score, which is defined as the harmonic mean of precision and recall:

(4) $$F_{1}=\frac{2P\times R}{P+R}$$

High precision relates to a low false positive rate, and high recall relates to a low false negative rate. High scores for both show that the classifier is returning accurate results (high precision), as well as returning a majority of all positive results (high recall). We did not try to optimize the feature set as the current is not the focus of this work (in Matlab, feature extraction takes one line of code: c = 10log10 (pwelch(x, 256, 192, 256, 4000)); % Include main body movement).

**Figure 8:**
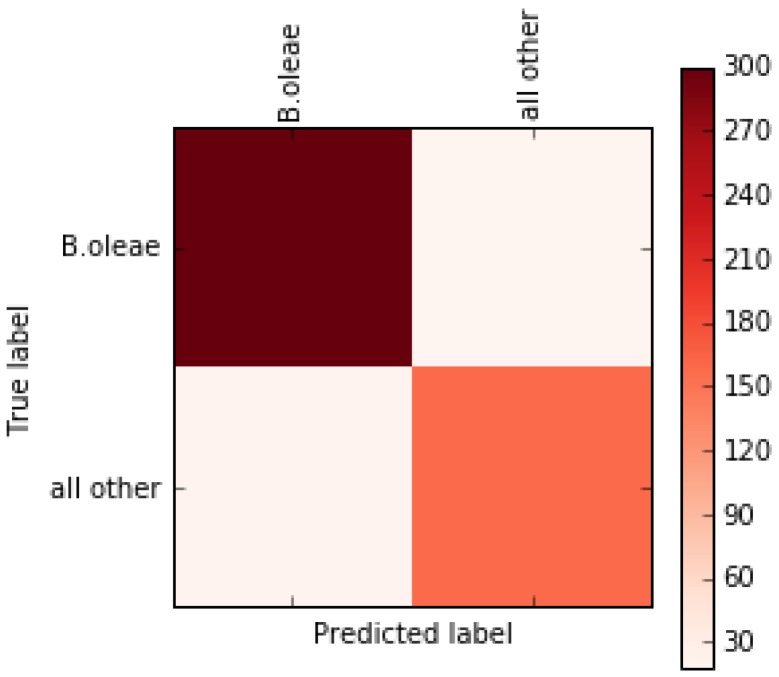
Confusion Matrix on a randomly selected 20% hold out set. Out of 319 cases of *B. oleae* 300 are classified as such and 19 are misclassified. From 176 cases of non-target fruit flies (i.e., *C. capitata*, *L. aristella, Drosophila*) 154 cases are correctly classified as non-target whereas 22 cases are False alarms. One can see clearly the diagonal structure of the confusion matrix indicating relatively low confusion rates.

We subsequently derive the confusion matrix of a classification model using a single, random peak of a 20% holdout set (see Table 3 and Figure 5). The confusion matrix, in our case (see also Table 4 and Figure 8) reveals the extent of misses and false alarms. One can notice the diagonal structure of the confusion matrix indicating that the strong tendency is to classify correctly the species and genera of the flying insect. The results in Table 4 are based on a Random Forest Classifier.

### 3.2. Verification Results in the Field

We placed the trap in the field, and left it exposed to the summer sun of Greece while performing several preliminary tests. One should note that the shift from the controlled environment to the field must be made gradually and with care due to the uncontrolled and often unforeseen situations of the field exposure. With the application of the infrared blocking film, the trap did not auto-trigger because of solar radiation, even when exposed directly to the mid-day sun that raised the internal temperature of the trap to 60 °C. Liquid attractants caused several problems and were replaced with gel-type ones along with Vapona insecticide stickers (i.e., dichlorvos). Careful regulation of the triggering threshold prevents the trap of registering counts due to moving branches and leaves of the trees providing shade to the trap that initially did cause false alarms. The trap is immune to smooth movements e.g., due to a gentle breeze but is vulnerable to abrupt hits or shocks that registered false alarm measurements in a windy day. 

## 4. Discussion

The experimental results from this work support the following suggestions: The in-situ, rule-based approach is compatible with pheromone attractants and has the advantage of being robust against temperature variations. *B. oleae* is active in the range 15–35 °C and its wingbeat frequency can vary from 170 Hz to 230 Hz within the same temperature range. By including the energy of the whole bandwidth in our calculations of a proper threshold, our approach is robust against temperature variations. Based on the need to push the verification accuracy further we examined the possibility of classifying the records using state-of-the-art classifiers that are computational intensive and require the snippets to be transmitted in order to be classified off-line on a server. Note that these recordings were all taken around 30 °C. In real-time operational conditions one will need classifiers that are immune to wingbeat changes due to temperature variations between 15 and 35 °C. We are indeed able to compensate efficiently for the effect of temperature variation, but this will not be discussed in this work. One should note that this data set reflects a worst case scenario where all insects flying into the trap are fruit flies and one only needs to spot the *B. oleae*. Still, the results are quite promising reaching a mean accuracy of ~91% (Table 3). The inclusion of the low frequency part due to body movement increased the accuracy by 1%. This is attributed to the fact that different species have morphological differences. All machine learning techniques achieve comparable results and we further elaborated on precision and recall scores (i.e., quantification of the miss and false alarm errors) in Table 4 and in Figure 8. The offline classification results are very encouraging and suggest that for a monitoring application it would be wise to consider the possibility of transmitting 1024 numbers per recording to the tracking site and classify them there and subsequently update the infestation maps on the server. There is room for improving the recognition scores by blending classification models and optimizing the feature extraction process. In order to operate the device in the field we follow two different routes: (a) we cover the trap with a semi-transparent infrared blocking film so that the infrared radiation of the sun is blocked from functioning as an emitter and, therefore, projecting any movement outside the trap (e.g., originating from moving tree branches, insects flying outside the trap etc.) to the sensor; (b) we investigate low-power versions of modulation/ demodulation circuits as in [13] that allow operating without the application of an infrared blocking film.

We suggest that automatic monitoring traps can be used to pinpoint or even predict the onset of infestations and help decision-making for properly regulated insecticide application. This technology is more expensive than a typical plastic trap. Note also that, although monitoring helps in adopting preventive measures to reduce damage, it cannot enforce eradication of the pest. Therefore, the cost to benefit ratio needs to be calculated objectively and at large scales before automatic monitoring is accepted. The annual losses due to this pest can be calculated objectively because they leave a mark on the olive fruits and they increase the acidity of olive oil that can be measured precisely. The losses that are reported annually and are attributed to this pest persuades us that automatic monitoring will finally prevail.

## 5. Conclusions

Electronic insect traps that monitor insects of economic importance such as fruit flies support the effort to ensure food supplies for a rapidly growing global population. We have demonstrated an autonomous trap that can obtain quality recordings of the wingbeat of flying-in insects, count them, discern species and transmit counts through the mobile network. Species verification can be achieved either in-situ or by transmitting the recordings and performing recognition on a server. As the version of the trap described in this paper does not transmit the recordings we derived recognition scores based on the recordings stored in the embedded SD card. When performing a decision in situ on the identity of the incoming insect it can only discern fruit flies against insects with great differences in size and/or spectrum. It cannot distinguish between different fruit flies (e.g., the difference between *B. oleae* and *C. capitata*), however, based on the recordings that are stored inside the trap and with a view to transmitting the recordings, the recognition scores are greatly improved, suggesting that transmission of the snippets allow for better discrimination, even among fruit flies, at the cost of an increased power consumption and decreased algorithmic complexity at the trap level.

## Figures and Tables

**Table 1:** Timing of events in CPU.

Process	Time
Collect data	200 ms
Copy data to buffer	800 μs
4×FFT (256 points)	7 ms
Log_10_	800 μs
Decision	1.2 ms
Store in SD	60 ms
**Total**	269.8 ms

**Table 2:** Dataset composition.

Insect	#Rec
*B. oleae*	913
*C. capitata*	623
*Drosophila*^+^	166
*L. aristella*	771
**Total**	2473

+ Genus *Drosophila*, species unidentified.

**Table 3:** *B. oleae* verification results.

Classifiers	%Mean Acc./Std
Linear SVC ^1^	88.46/1.24
RBF SVM ^2^	90.52/0.99
RF ^3^	91.05/1.55
ADABOOST	88.62/1.01
X-TREE ^4^	91.13/1.21
GBC ^5^	91.63/1.31
CNN ^6^	90.40/1.18

^1^ linear kernel, C = 0.01; ^2^ radian basis function kernel, gamma = 0.009, C = 0.2; ^3,4^ #trees = 650, min_samples_split = 2, min_samples_leaf = 1; ^5^ min_samples_split = 5, min_samples_leaf = 30, max_depth = 4; ^6^ SGD optimizer (learn_rate = 0.01, decay = 1 × 10^−4^, epochs = 60). Mean accuracy of top-tier classifiers using a 10-fold cross validation scheme with 20% of the corpus holdout. Verification results of *B. oleae* (913 cases) against three other fruit flies (1560 cases). Note that each species contains both sexes. Mean and standard deviation of accuracy measure over all folds (% mean/std over). Linear SVC: Linear Support Vector Classifier, RBF SVM: Radial Basis Function Support Vector Machine, RF: Random Forests, Adaboost: Adaboost Meta classifier, X-Trees: Extra Randomized Trees, GBC: Gradient boosting Classifier, CNN: 1D 3 layers, Convolutional Neural Network.

**Table 4:** Different accuracy metrics using a 20% hold out set.

Species	Random Forest Classifier			
	Precision	Recall	*F*_1_	#Rec
*B. oleae*	0.96	0.94	0.95	319
*All other*	0.90	0.92	0.91	176
Avg/total	0.93	0.93	0.93	495

## Supplementary Materials

Video S1: Automated surveillance of fruit flies, is available online at https://www.youtube.com/watch? v=IdWVaCyHEVI.

## Appendix A

The cost is, of course, subject to change but in order to help with the assessment of the cost/benefit tradeoff of this new technology we present a detailed cost breakdown and an item list in Table A1.

**Table A1:** Cost breakdown as per 10 September 2016 in Euros.

Item	Part Number	Qty/Board	Price/Board		
			1	100	1000
Emitter	SFH4356	20	13.36	3.58	3.36
Receiver	TEMD5110X01	13	12.4	7.48	6.34
Microcontroller	MSP432P401R	1	9	5	3.58
Temperature/RH Sensor	Si7021	1	3.98	3.19	2.87
GSM/GPS	SIM908	1	22	17	15
Other Electronic Components	ICs, Capacitors, Resistors, Connectors, PCBs		15	11	7
Plastic parts	Receiver & Transmitter housing, McPhail trap, add-on kit, diffuser		5	4	3
Battery	SAMSUNG IRCI18650-32A	2	14	11	8
**Total (€)**			**94.74**	**62.25**	**49.15**

SD not included in the price-list as it is included only in the research-version of the trap.
